# Bee Venom Activates the Nrf2/HO-1 and TrkB/CREB/BDNF Pathways in Neuronal Cell Responses against Oxidative Stress Induced by Aβ_1–42_

**DOI:** 10.3390/ijms23031193

**Published:** 2022-01-21

**Authors:** Cong Duc Nguyen, Jaehee Yoo, Sun-Young Hwang, Sung-Young Cho, Myeonghun Kim, Hyemin Jang, Kyoung Ok No, Jeong Cheol Shin, Jae-Hong Kim, Gihyun Lee

**Affiliations:** 1 College of Korean Medicine, Dongshin University, 67 Dongshindae-gil, Naju 58245, Korea; ducngcong@dsu.ac.kr (C.D.N.); tigger5368@naver.com (S.-Y.H.); tjddud1220@nate.com (S.-Y.C.); nevertmxk@dsu.ac.kr (M.K.); persona@dsu.ac.kr (H.J.); nko1386@dsu.ac.kr (K.O.N.); 2 Department of Acupuncture and Moxibustion Medicine, Dongshin University, 67 Dongshindae-gil, Naju 58245, Korea; skfl4747@dsu.ac.kr (J.Y.); fire-sjc@hanmail.net (J.C.S.); 3 Dongshin University Gwangju Korean Medicine Hospital, 141 Wolsan-ro Nam-gu, Gwangju 61619, Korea; 4 Dongshin University Mokpo Korean Medicine Hospital, 313 Baengnyeon-daero, Mokpo 58665, Korea

**Keywords:** bee venom, BDNF, amyloid-beta, Nrf2, neurodegeneration

## Abstract

Honeybee venom has recently been considered an anti-neurodegenerative agent, primarily due to its anti-inflammatory effects. The natural accumulation of amyloid-beta (Aβ) in the brain is reported to be the natural cause of aging neural ability downfall, and oxidative stress is the main route by which Aβ ignites its neural toxicity. Anti-neural oxidative stress is considered an effective approach for neurodegenerative therapy. To date, it is unclear how bee venom ameliorates neuronal cells in oxidative stress induced by Aβ. Here, we evaluated the neuroprotective effect of bee venom on Aβ-induced neural oxidative stress in both HT22 cells and an animal model. Our results indicate that bee venom protected HT22 cells against apoptosis induced by Aβ_1–42_. This protective effect was explained by the increased nuclear translocation of nuclear factor erythroid 2-like 2 (Nrf2), consequently upregulating the production of heme oxygenase-1 (HO-1), a critical cellular instinct antioxidant enzyme that neutralizes excessive oxidative stress. Furthermore, bee venom treatment activated the tropomyosin-related kinase receptor B (TrkB)/cAMP response element-binding (CREB)/brain-derived neurotrophic factor (BDNF), which is closely related to the promotion of cellular antioxidant defense and neuronal functions. A mouse model with cognitive deficits induced by Aβ_1–42_ intracerebroventricular (ICV) injections was also used. Bee venom enhanced animal cognitive ability and enhanced neural cell genesis in the hippocampal dentate gyrus region in a dose-dependent manner. Further analysis of animal brain tissue and serum confirmed that bee venom reduced oxidative stress, cholinergic system activity, and intercellular neurotrophic factor regulation, which were all adversely affected by Aβ_1–42_. Our study demonstrates that bee venom exerts antioxidant and neuroprotective actions against neural oxidative stress caused by Aβ_1–42,_ thereby promoting its use as a therapeutic agent for neurodegenerative disorders.

## 1. Introduction

Maintaining the yin-yang balance has been a holistic approach in Korean medicine and traditional East Asian medicine to foster stable health, and a tilt in this stability would emerge as a disease condition in the human body [1]. Oxidative stress produced in the body, as residues from natural metabolism, constantly plays a damaging role in cellular regulation. In intrinsic neuro-aging, oxidative stress caused by naturally deposited Aβ in the human brain is considered the main cause of neurodegeneration [2,3]. If we could symbolize this damaging factor as the yin haft; then the cellular intrinsic antioxidant defenses, usually reduced in neurodegenerative disorders [4,5,6], can be considered the opposite yang factor. Targeting the upregulation of endogenous antioxidant enzymes to neutralize excessive oxidative stress and invigorate neural cell redox balance has become a well-established approach to slow down the progression of neurodegenerative diseases [5,6,7,8]. This strategy interestingly reflects part of the East Asian medicine philosophy, a holistic therapy is not to focus on interacting directly with the disease itself but to reinforce the weakened half in the yin-yang balance to repel the illness by regaining energy harmony [9]. The present study on bee venom and its antioxidant properties in neurodegeneration provides an example of this focal point in Eastern and Western medical philosophy.

Bee venom medicine has long been used in Korea to relieve pain symptoms and treat inflammatory diseases. Recent clinical and experimental studies have demonstrated that bee venom and its derived active components are applicable to a wide range of neurodegenerative disorders. The effects of bee venom on neural disease are known primarily through the modulation of inflammatory responses [10].

It has been well established that neuro-oxidative stress ignited by naturally produced Aβ deposited in the aging brain is a main factor in the development of neural disorders [11,12]. Aβ intracerebroventricular (ICV) injection into healthy mice has become a tool that mimics natural dementia [13,14,15,16,17,18]. Aβ aggregates were discovered to form ion-like channels in cell membranes, thus encouraging Ca^2+^ influx and destabilizing intercellular electrochemical balance [19,20,21]. Aβ was also discovered to produce mitochondrial malformations via the inactivation of mitochondrial complexes I and IV [22,23]. Metal ions, such as copper, can stimulate the production of further oxidative stress when bound to Aβ [24]. In neurodegenerative progression, the overproduction of reactive oxygen species (ROS) from these central occurrences interferes with cellular redox harmony, disrupts biochemical pathways. As a result, increases MDA, LDH, and protein carbonyl levels, eventually initiates neuroinflammation [25], neuron cell death, and promotes neurocognitive impairments [26,27,28]. 

Recent studies have indicated that antioxidant properties may provide a more holistic strategy to the anti-inflammatory approach. Antioxidant and detoxification genes are believed to preserve cellular homeostasis and eliminate toxins before they can cause damage and activate inflammatory responses [29,30,31]. Nuclear factor erythroid 2-like 2 (Nrf2) is a transcription factor that regulates heme oxygenase-1 (HO-1) expression, a crucial antioxidant enzyme that plays a major role in neutralizing oxidative stress. Several studies have demonstrated that Nrf2 contributes to the anti-inflammatory process by orchestrating the recruitment of inflammatory cells [32,33]. Therefore, the identification of novel Nrf2 activators has become a key point in drug discovery to combat neurodegeneration [4,6,7].

Although previous studies have depicted bee venom as a potent anti-neurodegenerative agent owing to its anti-inflammatory ability [10], the primitive progression of neuronal damage is due to oxidative stress rather than inflammation [29,30,31,33]. Previous cardiology and renal studies have suggested that melittin, the major component, takes up more than half of bee venom’s dry weight and reinforces cellular redox balance under oxidative stress by stimulating the nuclear translocation of Nrf2 and upregulating HO-1 expression [34,35]. In addition, the proficiency of bee venom to normalize depleted HO-1 production was mentioned in other steatohepatitis [36], wound healing [37], and hepatitis [38] studies, but it has not been examined in anti-neuro-disorder research.

Therefore, we propose that bee venom’s neuronal protective effects might be accountable due to its potential to buttress cellular redox balance by upregulating cellular instrinsic antioxidative stress barriers. Clarifying this uncharted aspect of bee venom mechanisms plays a key role in promoting this drug candidate as a holistic and effective neurodegenerative agent.

## 2. Material and Methods

### 2.1. Materials

Dulbecco’s Modified Eagle Medium (DMEM), PBS, and TBS were purchased from Welgene Inc. (Gyeongsan, Gyeongbuk, Korea). Trypsin-EDTA (0.25%), FBS, 2,7-dichlorofluorescein diacetate (DCFDA), and penicillin–streptomycin were obtained from Invitrogen (Carlsbad, CA, USA). Bee venom from *Apis mellifera*, Aβ_1–__42_, and Fluoromount™ Aqueous Mounting Medium were purchased from Sigma–Aldrich (St. Louis, MO, USA). The Quanti MAX^TM^ WST-8 Cell Viability Assay Kit was purchased from BIOMAX (Seoul, Korea). The specific mouse primary antibodies (TrkB, p-TrkB, Bax, Bcl-2, 1-HO, Nrf2, CREB, p-CREB, iNOS, and β-actin) and secondary anti-mouse IgG were purchased from Cell Signaling Technology, Inc. (Danvers, MA, USA). BDNF, mAChR1, and lamin B antibodies were purchased from Merck KGaA (Darmstadt, Germany). The Pro-PREP protein extraction solution was purchased from iNtRON Biotechnology (Seoul, Korea). The first doublecortin antibody from Santa Cruz Biotechnology (Dallas, TX, USA) and Alexa Fluor 488 secondary antibody (Thermo Fisher Scientific, Waltham, MA, USA) were used. Cellular ROS, superoxide dismutase (SOD), lipid peroxidation (MDA), lactate dehydrogenase (LDH), and protein carbonyl activity required acetylcholine esterase (AChE) assay kits, AIF first antibody, CytoC first antibody, and CleaCas3 first antibody, annexin V, and propidium iodide (PI) from Abcam (Cambridge, UK). A nuclear extraction kit was purchased from Cayman Chemical Company, Inc. (Ann Arbor, MI, USA). An acetylcholine (Ach) level assay kit was purchased from Cell Biolabs (San Diego, CA, USA).

### 2.2. Cell Culturing

The mouse hippocampal HT22 cell line was a gift from Professor Na Chang Su, College of Korean Medicine, Dongshin University. Cells were cultured in DMEM supplemented with FBS (10%) and penicillin–streptomycin (1%). The incubation conditions were 5% CO_2_ and 37 °C in 96-well plates. The cells were cultured with the following conditions: 1 × 10^4^ cells per well in 0.1 mL media, and 6-well plates with 3 × 10^5^ cells per well in 3 mL media. Twenty-four hours after cell seeding, further drug administration was carried out as described in the following sections.

### 2.3. Cell Viability Assay and Drug Administration

To assess cell viability after seeding for 24 h in 96-well plates, bee venom was added as follows: 0.15–50 μg/mL for a safety concentration test; for the cellular protection activity, 0.15–5 μg/mL, and 1 h later, 5 μM of Aβ_1–42_ was added. At different time points—0 h, 4 h, 8 h, 12 h, 16 h, 20 h, and 24 h after Aβ_1–42_ was added—cell availability was measured by adding 100 μL WST-8 and incubating for 1 h. Absorbance at 450 nm was measured using a Versa Max Microplate Reader (Molecular Devices, San Jose, CA, USA). The experiments were repeated three times, and 2 mM Triton X was used to achieve absolute cell death as a reference for the availability percentage calculation.

### 2.4. Cell Apoptosis and ROS Levels Assessment by Flow Cytometry

In 6-well plates, the experiment was performed 8 h after treatment with Aβ_1–42_. All cells (floating and attached to the well bottom) were harvested and washed twice with PBS. Cells (1 × 10^5^) were collected and resuspended in 500 µL of 1× binding buffer. The staining process was carried out by adding 5 µL of annexin V (detected by the FITC channel) and 5 µL of propidium iodide (PI, detected by the phycoerythrin (PE) channel). For ROS detection, 500 µL of 25 µM DCFDA (detected by the FITC channel) was used. The cells were incubated at 37 °C for 15 min in the dark. Approximately 10,000 cells from each sample were used to produce the data. Analysis was carried out using a Cytoflex flow cytometer (Beckman Coulter, Inc., Brea, CA, USA.) according to a previous study protocol [39].

### 2.5. Measurements of In Vitro Cell Apoptosis, Cell ROS, SOD, MDA, LDH, and Protein Carbonyl

For apoptosis, and ROS immunofluorescence imaging, the experiment was performed 8 h after treatment with Aβ_1–42_ in black 96-well plates with clear bottoms. For the apoptosis assay, the cells were washed once with 200 µL assay buffer, after which 1 µL of CytoCalcein 450 solution was added (for live cell labeling, Ex/Em = 405/450 nm) + 1 µL of 7-AAD solution (for late apoptosis and dead cells labeling, Ex/Em = 550/650 nm), + 2 µL of Apopxin Green Indicator solution (for detecting apoptotic cells, Ex/Em = 490/525 nm), and incubated at room temperature in the dark for 45 min. Subsequently, the cells were washed once, and the wells were filled with 100 µL assay buffer and observed under a 50× objective. For the cellular ROS assay, the cells were washed twice with assay buffer, and then each well was filled with 200 µL of 25 µM DCFDA solution (Ex/Em = 490/525 nm) and incubated at 37 °C in the dark for 15 min. The DCFDA solution was then removed and replaced with 100 µL of assay buffer and observed with a 30× objective. An Invitrogen EVOS FL Auto Imaging System fluorescence microscope (Thermo Fisher Scientific, Waltham, MA, USA) was used.

To measure SOD, MDA, LDH, and protein carbonyl levels in 6-well plates, the experiment was performed 8 h after treatment with Aβ_1–42_; cell lysate and assay performance were carried out according to the manufacturer’s instructions. The data were collected using a VersaMax microplate reader (Molecular Devices, San Jose, CA, USA).

### 2.6. Preparation of Whole-Cell, Cytosolic, and Nuclear Proteins

Whole-cell, nuclear, and cytosolic proteins were collected to study the nuclear translocation of Nrf2 [40]. In a 6-well plate, 8 h after Aβ_1–42_ treatment, cells were harvested. Part of the cell pellet was lysed immediately to collect whole-cell proteins. The remaining part was added to a hypotonic buffer containing phosphatase and protease inhibitors and incubated for 10 min at 4 °C. Subsequently, the 10% Nonidet P-40 assay reagent was introduced to facilitate phase separation. Centrifugation was performed (14,000× *g*, 30 s), and supernatant with cytosolic content was carefully collected and stored at −80 °C. The remaining mixture was introduced into the nuclear extraction buffer and incubated for 30 min at 4 °C. This was then centrifuged (14,000× *g*, 10 min), and the supernatant was collected as the nuclear extract. The blot membranes of cytosolic and nuclear proteins were processed similarly, and the imaging exposure time was used to express the true scale of protein expression between nuclear and cytosolic proteins. To confirm the accepted separated nuclear and cytosolic contents, the total cytosolic lamin B signal must be less than 5% of that in the nuclear extract. Similarly, the total cytosolic β-actin signal must be less than 5% of that in the nuclear extract.

### 2.7. Animals

Male ICR mice (7 weeks old) were purchased from Samtaco (Gyeonggi-do, Korea) and housed with a maximum of five mice per cage under pathogen-free conditions (22–26 °C; humidity: 50–60%), with a 12-h light/dark cycle and free access to standard food (Sangyang Co., Osen, Korea) and water. Acclimation was carried out for five days before the experiments were started. Behavioral experiments were conducted in accordance with the Guide for Care and Use of Laboratory Animals of the National Research Council (NRC, 1996) and were approved by the Committee of Animal Care and Experiment of Dongshin University, Korea (DSU2019-04-02).

### 2.8. In Vivo Drug Administration

To stimulate aggregation, 1 mg Aβ_1–42_ was dissolved in 100 mL sterile physiological PBS in a tube, which was sealed and incubated for four days at 37 °C [41]. After anesthesia, Aβ_1–42_ ICV injections were performed as described, and the prepared Aβ_1–42_ solution (3 µL, containing 3 µg aggregated Aβ_1–42_), was injected into the right cerebral ventricle using a 28-gauge needle with stereotaxic coordinates (in mm), A: −0.22, L: 1.0, V: 2.5, from the bregma, flow rate of 3 μL/5 min [17,42,43]. On day 1, except for the naïve group that received 3 µL of PBS, all mice were injected with 3 µL of a solution containing 3 μg of Aβ_1–42_. A bee venom maximum safe dosage of approximately 13.19 mg/kg was previously determined [44]. Another study applied 0.1 mg/kg of bee venom into a mouse vascular neurodegenerative model [45]. Therefore, we used 0.1 mg/kg of bee venom as well as a second dosage of 1 mg/kg to study any possible dose-dependent effects. Bee venom was administered via a 50 μL subcutaneous (SC) injection at a non-acupoint—the hypochondrium, 10 mm above the iliac crest [46]—to avoid interference from acupuncture effects. Bee venom injections were administered on days 3, 5, 7, 9, and 11. The mice were categorized into the following groups according to treatment (*n* = 6):

Group 1—PBS ICV + PBS SC;

Group 2—3 μg Aβ_1–42_ ICV + PBS SC;

Group 3—3 μg Aβ_1–42_ ICV + bee venom 0.1 mg/kg SC;

Group 4—3 μg Aβ_1–42_ ICV + bee venom 1 mg/kg SC.

### 2.9. Morris Water Maze (MWM)

The spatial learning and memory capacity of the mice were assessed by MWM as previously described, with minor modifications [47]. Briefly, a circular, black-painted water tank (diameter: 120 cm; height: 50 cm) surrounded by white visual cues—a star, square, rectangle, and circle—was used. The temperature was maintained at 25 ± 2 °C. The tank was separated into four quadrants, and a black platform (diameter, 10 cm; height, 25 cm) was in the northwest quadrant. Each time a mouse was introduced to the water maze experiments, if it was unable to find the platform when time had run out, it would be gently guided to the platform by hand and moved back to the cage, which was always kept warm at 32 °C. The experimental procedure included 3 subsequent segments. (1) Adaptive training: On day 6, the animals were left to swim for 100 s in the tank to find the visible platform 1 cm above the water, three times a day; (2) Escape training: On days 7–10, mice were left to swim for a maximum of 100 s to find the platform that was submerged 1 cm below the water, two times a day;(3) Probe test: Immediately after the last hidden platform test on day 10, the platform was removed and each mouse was left to swim freely for 120 s to observe their swimming behavior, performed only once for each mouse.

The ANY-maze (Stoelting Co., Wood Lane, IL, USA) animal behavior monitoring software was used to record the indexes and form a distribution heat map for the last probe test.

### 2.10. Collection of Animals Hippocampus and Blood Samples

After the MWM experiment, on day 11, all mice were anesthetized, their chests were opened, and blood was collected from the hearts. After collection, whole blood was allowed to clot by leaving it undisturbed at room temperature for 40 min. Subsequently, serum was separated by centrifugation at 3000 rpm for 10 min at 4 °C. Hippocampuses were also collected from the brains immediately. Within the groups, the hippocampuses of a subset of animals were used for immunofluorescence analysis (these animals were perfused with ice-cold 4% paraformaldehyde, and their hippocampuses were fixed with 4% paraformaldehyde overnight at 4 °C); the hippocampuses of the other animals were immediately subjected to biochemical or Western blot analysis the same day.

### 2.11. Doublecortin Immunofluorescence Staining

Post-fixed brains were incubated with 30% sucrose in PBS for 1 d at 4 °C, which was repeated once with 30% sucrose in PBS solution. The brains were frozen using dry CO_2_ ice powder and sliced into 30-μm sagittal sections before being transferred to gelatin-coated slides [48]. At room temperature, the sections were incubated with 5% bovine calf serum (1 h) and, subsequently, with doublecortin primary antibody (1:100, 2 h), They were rinsed in PBS twice for 15 min each, followed by incubation with Alexa Fluor 488 secondary antibody (Ex/Em = 490/525 nm, 1:200, 2 h) and two additional PBS rinses for 15 min each. The samples were topped in Fluoromount™ Aqueous Mounting Medium and covered with glass coverslips for microscopic imaging. The data were collected using the Invitrogen EVOS FL Auto Imaging System (Thermo Fisher Scientific, Waltham, MA, USA) with a 30× objective. The number of doublecortin-positive stained neurons was counted in the same area of three slides per animal and three animals per group.

### 2.12. Western Blot Analysis

For the in vitro experiments, cells were grown in 6-well plates, and after 8 h of Aβ_1–42_ treatment, cell pellets were collected and lysed. For the in vivo samples, the mouse hippocampuses were homogenized with lysate buffer immediately after collection and proceeded to the next steps on the same day. Western blotting was performed as described previously [49]. The Amersham™ ImageQuant™ 800 biomolecular imager system (General Electric, Boston, MA, USA) was used to obtain protein signals. ImageJ software (National Institutes of Health, Bethesda, MD, USA) and the command directory “Process/Binary/Make Binary” were used to convert the protein band images into a binary version and then analyzed to collect signal intensities.

### 2.13. Measurements of ROS, MDA, NO, AchE, and Ach Levels in In Vivo Samples

Hippocampal lysate or serum samples were prepared, and assays were carried out according to the manufacturer’s instructions. Data were collected using a VersaMax microplate reader (Molecular Devices, San Jose, CA, USA).

### 2.14. Statistical Analysis

Data are presented as the mean ± standard deviation (SD) for each group. Differences in quantitative measurements were assessed by one-way analysis of variance followed by the Kruskal–Wallis test using Prism software version 8.1. The number of repetitions was given in this method. Differences were considered significant with *p* < 0.05.

## 3. Results

### 3.1. Bee Venom Exhibits Protective Effects on HT22 Cells against Cytotoxicity Induced by Aβ_1–42_

**Figure 1:**
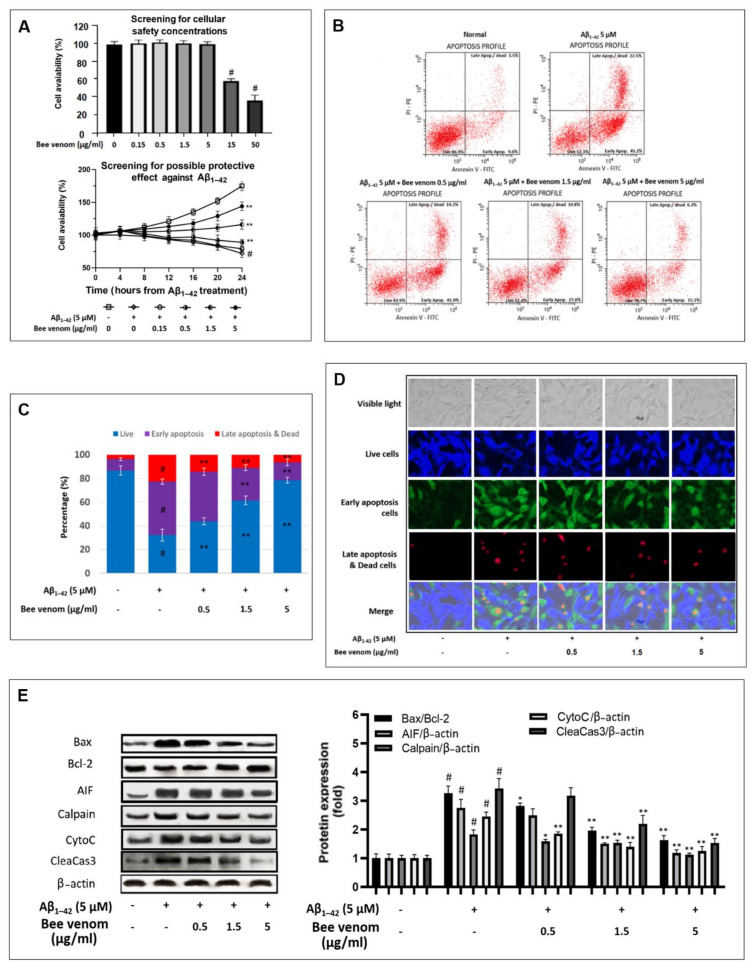
Bee venom protected HT22 cells against Aβ_1–42_ induced apoptosis (**A**) Experiments screened for cellular safety concentration and possible protective effect against Aβ_1–42_-stress induced in HT22 cells. First experiment—screening for bee venom cellular safety. The results indicate that 5 μg/mL was the highest concentration that did not significantly alter HT22 cell availability after 24 h of incubation (bee venom was pre-treated and cell viability was examined after 24 h). For protective effects of bee venom against Aβ_1–42_ stress. The results indicate that 0.5–5 μg/mL bee venom dose-dependently ameliorated HT22 cell availability (bee venom was introduced 1 h before 5 μM Aβ_1–42_ challenge, cell availability was examined every 4 h for one day). (**B**) Apoptosis-inhibitory effects of bee venom on Aβ_1–42_ induced HT-22 cells by flow cytometry analysis with (**C**) summarized statistics results. The results indicate that 0.5 to 5 μg/mL of bee venom dose-dependently ameliorated HT22 cell apoptosis under Aβ_1–42_ (5 μM) stress-induced conditions (8 h after Aβ_1–42_ (5 μM) challenge, cells underwent immunofluorescence staining, and cyto flowmetery analysis was carried out). (**D**) Fluorescence microscopy analysis of the apoptosis level of cells from different treatment groups, an imaging supplement to parts (**B**,**C**) (8 h after Aβ_1–42_ (5 μM) challenge, cells underwent immunofluorescence staining and analysis). (**E**) Western blot analysis of key apoptosis proteins. The results indicate that bee venom (0.5–5) μg/mL dose-dependently stabilized the expression of pro- and anti-apoptosis proteins under Aβ_1–42_ stress challenge (8 h after Aβ_1–42_ (5 μM) challenge, cells were lysed, and Western blot was performed). Data are shown as mean ± SD in triplicate. # *p* < 0.01 vs. control; * *p* < 0.05 or ** *p* < 0.01 vs. Aβ_1–42_ only-treated group.

To begin the experiment, we determined the cellular safety concentrations of bee venom. By testing bee venom concentrations of 0.15–50 μg/mL on HT22 cells and checking the cell availability after 24 h, we concluded that the concentration of 5 μg/mL is the maximum safe dosage (Figure 1A). Subsequently, for screening the protection effect of bee venom against Aβ_1–42_-induced cytotoxic in HT22 cells, 0.15–5 μg/mL bee venom was screened. As a result, bee venom at 0.5–5 μg/mL exhibited a dose-dependent effect on HT22 cell availability at 12 to 24 h after Aβ_1–42_ challenge; therefore, this dosage was selected for later in vitro experiments (Figure 1A). In addition, at 8 h after Aβ_1–42_ treatment, we recorded the cell viability of the Aβ_1–42_-only treated group as 98.5 ± 3.5%; this level is approximately 92.2% compared to that of the non-treatment HT22 cells group, whose cell viability was measured at 108.0 ± 3.9%. We decided to use the cells developed at this time point for further later analysis, rather than the 24 h time point after the Aβ_1–42_ treatment.

Cell apoptosis assays were conducted to further confirm the neuroprotective effects of bee venom. The results revealed that in a dose-dependent manner, bee venom at 0.5–5 μg/mL reduced apoptosis levels in HT22 cells that were under stress induced by Aβ_1–42_ (Figure 1B,D). Western blotting analysis indicated that the Bax/Bcl-2 protein ratio increased three-fold once cells were introduced with Aβ_1–42_ but decreased when treated with bee venom (Figure 1E). Other apoptosis-associated proteins, such as AIF, Calpain, CytoC, and CleaCas3 are also key apoptosis markers that have been studied in HT22 cells [40,50]. Bee venom had dose-dependently regulated the overexpression of these proteins, indicating a good anti-apoptosis effect.

### 3.2. Bee Venom Reduces Oxidative Stress Expressions of HT22 Cells during Aβ_1–42_ Challenge

**Figure 2:**
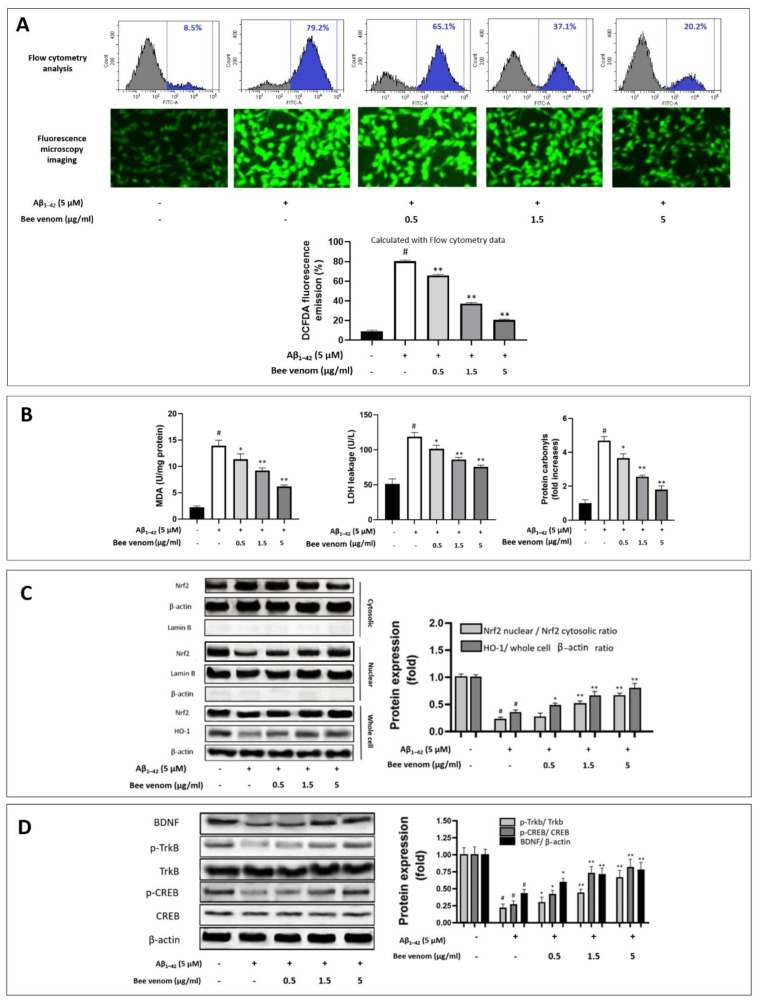
Bee venom upregulates Nrf2/HO-1 and BDNF/TrkB/CREB pathways in HT22 cell in Aβ_1–42_ induced stress (**A**) Flow cytometry and fluorescence microscopy imaging analysis to determine cellular ROS levels, and summarized statistics results calculated with flow cytometry data. The results indicated that bee venom (0.5–5 μg/mL) dose-dependently normalized the expression of oxidative stress under Aβ_1–42_ challenge (8 h after Aβ_1–42_ (5 μM) challenge, immunofluorescence analysis by DCFDA staining was performed). (**B**) Assay results of MDA, LDH, and protein carbonyls levels. The results indicate that bee venom (0.5–5 μg/mL) dose-dependently downregulated MDA, LDH, and protein carbonyl parameters under Aβ_1–42_ stress challenge (8 h after Aβ_1–42_ (5 μM) challenge, measuring kits for MDA, LDH, and protein carbonyls were applied to determine the parameters). (**C**) Effect of bee venom on nuclear translocation of Nrf2 and production of HO-1 antioxidant enzyme. The results suggest that bee venom (0.5–5 μg/mL) dose-dependently enhanced Nrf2 nuclear translocation and increased the expression of the antioxidant enzyme HO-1 in HT22 cells under Aβ_1–42_ (5 μM) challenge (8 h after Aβ_1–42_ (5 μM) challenge, whole-cell, cytosolic, and nuclear proteins were obtained, and Western blot was conducted). (**D**) Effects of bee venom on the activation of brain-derived neurotrophic factor signaling. The results suggest that bee venom enhanced the performance of the BDNF/TrkB/CREB pathway during Aβ_1–42_ (5 μM) challenge (8 h after Aβ_1–42_ (5 μM) challenge, cells were lysed, and Western blot was performed). Data are shown as mean ± SD in triplicate. # *p* < 0.01 vs. control; * *p* < 0.05 or ** *p* < 0.01 vs. Aβ_1–42_ only-treated group.

Aβ_1–42_ aggregations stimulate cellular redox imbalance, which is considered a main route of neurodegeneration disorder. Our experiments recorded a sharp rise in cellular ROS when HT22 cells were introduced with Aβ_1–42_, whereas bee venom (0.5 to 5 μg/1 mL) significantly reduced cellular ROS levels (Figure 2A). Further analysis of key oxidative markers showed that bee venom dose-dependently inhibited the overexpression of the important oxidative stress markers MDA and LDH. Protein carbonyl, an end-result indicator of a cellular oxidative stress damage reaction chain, is a specific oxidative stress marker in various diseases, including Alzheimer’s disease [51]. Our results indicate that 0.5–5 μg/mL bee venom significantly downregulated cellular protein carbonyl levels (Figure 2B).

### 3.3. Bee Venom Induces Intrinsic Antioxidant Regulation Nrf2/HO-1 of HT22 Cells against Aβ_1–42_-Induced Oxidative Stress Challenge

Damaging oxidative stress is widely considered a key factor in neuro-aging studies [24]. The ability of whole honeybee venom to regulate cellular antioxidant defense has not been well addressed in neurology. To explain the mechanisms by which this drug candidate shields HT22 cells against oxidative stress induced by Aβ_1–42_, we analyzed the upregulation of the Nrf2/HO-1 pathway under bee venom pre-treatment. Consistent with previous studies, Nrf2 nuclear translocation was significantly blocked in Alzheimer progression [4,5]. Our results indicate that Aβ_1–42_ treatment reduced Nrf2 migration into the cellular nucleus, resulting in the downregulation of HO-1 antioxidant enzyme production. In contrast, bee venom (0.5–5 μg/mL) pre-treatment dose-dependently enhanced Nrf2 nuclear translocation. At 5 μg/mL, bee venom treatment markedly increased Nrf2 nuclear translocation and HO-1 production by nearly three-fold compared to Aβ_1–42_ treatment alone (Figure 2C).

### 3.4. Effect of Bee Venom on the TrkB/CREB/BDNF Pathway

In neural cellular protective physiology, the TrkB/CREB/BDNF signaling pathway is known as the key regulator pathway in maintaining neurogenesis and cell functions and is significant for reinforcing Nrf2/HO-1 upregulation [6,28,40,52]. Bee venom dose-dependent treatment significantly recovered the phosphorylation of p-TrkB, p-CREB, and BDNF expression compared to Aβ_1–42_ treatment alone (Figure 2D). Taken together, our results show that bee venom demonstrated a potent neuroprotective effect in HT22 cells under Aβ_1–42_ stress induced via Nrf2/HO-1 upregulation and the TrkB/CREB/BDNF pathways.

### 3.5. Bee Venom-Enhanced Learning and Memory Function of Aβ_1–42_-Induced Cognitive Impairment in Mice Behavior Experiment

**Figure 3:**
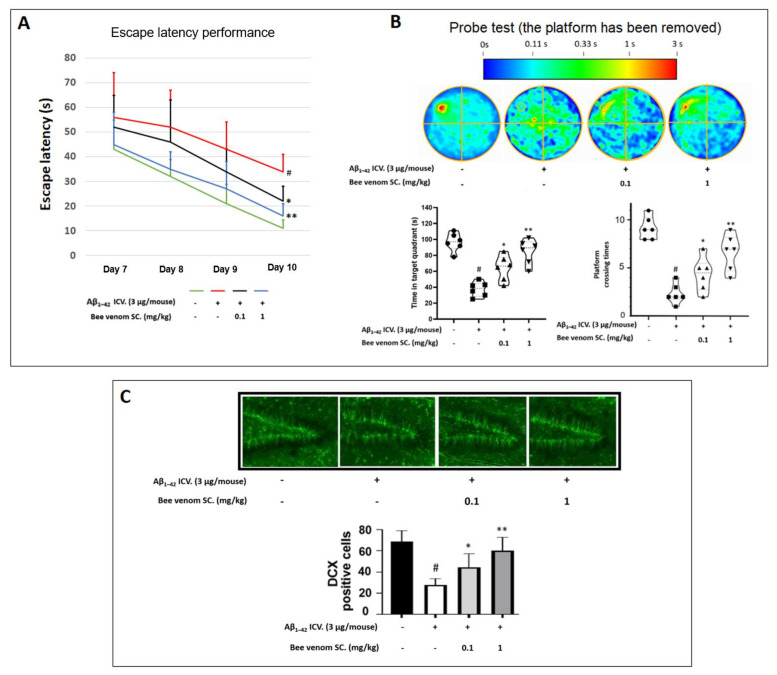
Bee venom recovers animal cognitive function and hippocampal neuronal development depleted by Aβ_1–42_ ICV pre-treatment (**A**) Protective effects of bee venom on memory in Aβ_1–42_ -treated mice studied in a Morris water maze experiment. Two tests (**A**,**B**) exhibited a decrease in learning and memory performance when induced with Aβ_1–42_ ICV, and bee venom (0.1–1 mg/kg) significantly reversed this cognitive dysfunction. (**A**) Animal escape latency performance on days 7 to 10. To conduct this experiment, mice were administered bee venom (0.1 or 1 mg/kg SC) after Aβ_1–42_ treatment (5 μg/mouse, ICV), an adaptive training was conducted on day 6, and the escape training test was performed on days 7 to 10. (**B**) A probe test was conducted at the end of day 10 to bolster behavior assessments (the platform was removed, and mice were allowed to swim freely for 120 s to ascertain their remembrance of the disappeared platform location). A color scale denotes the average allocation position of animals in each group. The circle positioned in the upper-left quadrant is the removed platform location. (**C**) Protective effects of bee venom on neuronal cells in the dentate gyrus region against Aβ_1–42_ induced stress. The results indicate that Aβ_1–42_ ICV treatment reduced neuron neurogenesis significantly, and this was drastically reversed by bee venom (brain sections were stained with doublecortin protein antibody, and the same areas of hippocampal dentate gyrus region were observed under a fluorescence microscope). Data are shown as mean ± SD in triplicate. # *p* < 0.01 vs. control; * *p* < 0.05 or ** *p* < 0.01 vs. Aβ_1–42_-only-treated group.

To mimic cognitive deficit in a mouse model, Aβ_1–42_ ICV injections were performed to induce an Alzheimer-like stage. MWM tests were sequentially carried out from days 7 to 10. Bee venom 0.1–1 mg/kg treatments significantly reduced the escape latency of mice on day 10 (Figure 3A), along with improved distribution in the target quadrant and the platform crossing time in the probe test session (Figure 3B) compared to Aβ_1–42_ treatment alone.

### 3.6. In Vivo Immunohistochemistry Study

Located inside the hippocampus, the dentate gyrus region is commonly studied thanks to its key role in the formation, recall, and discrimination of episodic memory [53]. The results indicate that Aβ_1–42_ ICV injection decreased the number of doublecortin-positive cells in the dentate gyrus by nearly three-fold, whereas bee venom injection (0.1 and 1 mg/kg) dose-dependently recovered this index (Figure 3C).

### 3.7. Oxidative and Inflammation Markers in Mouse Hippocampus and Serum

**Figure 4:**
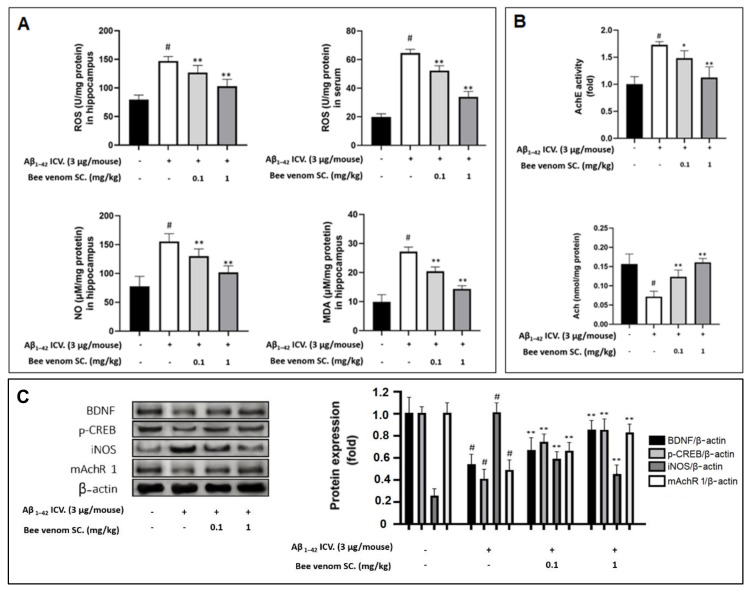
Bee venom regains mouse hippocampus biochemiscal stability of Aβ_1–42_ ICV pre-treatment mice (**A**) Major antioxidant parameters ROS, NO, and MDA were examined in in vivo samples: The results show that Aβ_1–42_ ICV treatment significantly increased oxidative stress in the hippocampus and serum, which was reversed by bee venom treatment (hippocampus extract and serum were prepared, and ROS, NO, and MDA levels were examined with respective measuring kits instructions). (**B**) Levels of AchE activity and Ach content in the mouse hippocampal. The cholinergic system exhibited amelioration against Aβ_1–42_ ICV-induced neurodegeneration as bee venom was administered (hippocampus extract were prepared, AchE and Ach levels were examined with respective measuring kits instructions). (**C**) Levels of BDNF, p-CREB, iNOS, and mAchR1 protein expression in mouse hippocampus. Bee venom dose-dependently exhibited protective effects against Aβ_1–42_-induced neuro-imbalance in vivo (Western blot analyses were conducted on protein extracts from hippocampal tissue). Data are shown as mean ± SD in triplicate. # *p* < 0.01 vs. control; * *p* < 0.05 or ** *p* < 0.01 vs. Aβ_1–42_-only-treated group.

Aβ_1–42_ injections significantly increased ROS, MDA, and NO levels in the hippocampal tissue and/or mouse serum. We observed that bee venom treatment ameliorated these ROS levels in a dose-dependent manner (Figure 4A). This proved the effectiveness of bee venom in downgrading the amount of damaging oxidative stress in vivo.

### 3.8. Study on In Vivo Cholinergic System

Neurodegeneration is characterized by a significant shortage of acetylcholine and key neuroreceptors, such as M1 muscarinic acetylcholine receptors (mAchR 1) [54], as well as an overexpression of acetylcholinesterase (AchE) [55]. In the hippocampal samples, we observed Aβ_1–42_ ICV injection resulted in approximately 40% loss in mAchR 1 expression (Figure 4C), a 1.6-fold increase in AchE activity, and a 70% decrease in ACh levels. However, they were all effectively stabilized by bee venom pretreatment (Figure 4B).

### 3.9. Effect of Bee Venom Treatment on the Expressions of Key Marker Proteins

Immunohistochemistry imaging revealed the capacity of bee venom to enhance hippocampal neurogenesis. We further confirmed this via the upregulation of key neurotrophic proteins, such as BNDF and p-CREB. As shown in Figure 4C, the results suggest that BNDF and p-CREB in the hippocampus were depleted in the Aβ_1–42_-only-treated group but were significantly upregulated in the bee venom-treated groups.

Inflammation is a crucial link that promotes neurodegeneration and is induced by Aβ_1–42_ [13,14,15,16,17,18]. In the previous section, the overexpressed hippocampal NO concentration was shown to be reduced by bee venom, which was confirmed by the reduction of iNOS protein expression due to bee venom, after being negatively altered by Aβ_1–42_ (Figure 4C).

## 4. Discussion

Natural aging neurodegenerative diseases, including Alzheimer’s disease, result in decreased human cognitive function, reduced human life span, and an overall decline in well-being [56]. The brain is sensitive to oxidative stress, and in comparison to other organs, the brain contains many oxidized unsaturated fatty acids and lipid peroxidation content. Regular brain activity also consumes higher oxygen per unit weight, and this nonstop metabolism constantly produces damaging oxidative stress [57].

During aging, Aβ is a byproduct of the degradation of the amyloid-beta precursor protein—an important functional protein that is concentrated in neuronal synapses. Accumulated Aβ naturally forms aggregates over time and alters the intercellular redox balance of neurons [24]. Neural cellular physiological processes, including defensive mechanisms and neurogenesis signaling activities, are regulated by electrophiles and ROS. Current trends strongly consider imbalanced ROS and excessive electrophiles as leading triggers of neural cell injury, whereas oxidative stress is crucial for the onset and progression of chief cerebrovascular and neurodegenerative disorders. These include Alzheimer’s disease, Parkinson’s disease, amyotrophic lateral sclerosis, Huntington’s disease, stroke, and aging. Nrf2 activation and nuclear translocation are decreased in neurodegenerative disorders. Nrf2 is the key modulator of the xenobiotic-activated receptor and is responsible for stimulating the antioxidative response pathway, neutralizing excessive oxidative stress, and maintaining cellular redox balance. Overwhelming oxidative stress and the defeat of the cellular intrinsic antioxidant defense barrier subsequently results in neuroinflammation [29,30,31,33]. Therefore, a holistic approach exists if a drug candidate exhibits neural cell Nfr2/HO-1 upregulation [4,5].

Previous cardiology and renal studies have suggested that melittin, the major component, makes up more than half of bee venom’s dry weight, stimulates the nuclear translocation of Nrf2 [34,35], and further upregulates HO-1 expression. Bee venom has proven itself in upregulating the expression of BDNF via activation of ERK [45], which suggests a positive effect on the TrkB/CREB/BDNF pathway, which promotes neural cell health and is key to reinforcing the regulation of cellular intrinsic antioxidant defense [6,57,58,59]. Therefore, bee venom has great potential for protecting neuron cells, such as HT22, against neurodegeneration promoted by Aβ-induced oxidative stress by regulating the Nrf2/HO-1 pathway, which is supported by the TrkB/CREB/BDNF pathway. In addition, a similar antioxidative protective effect might be observed in an Aβ-induced in vivo model, followed by a series of positive effects on the recovery of depleted cognitive memory, anatomical neurogenesis, neurotrophic factors, and the cholinergic system, as well as the normalization of an over-stimulated inflammation reaction.

We previously published an article that suggested melittin to upregulate both Nrf2/HO-1 and BDNF/TrkB/CREB, and slow down amyloid-beta neuro damage. Nrf2/HO-1 pathway normalization, is a strong pillar in anti-neurodegeneration strategies [4,5,7]. Knowing fully the characteristic of a medicine is important as it is the basement from which a razor-sharp clinical therapeutic plan can be developed. Therefore, even though melittin is the major component of bee venom, without a conducting proper study, it should not be taken for granted that bee venom can also upregulate Nrf2/HO-1 to protect neuro-disorder conditions.

As mentioned, the ability of bee venom to normalize depleted HO-1 levels and combat ROS production was mentioned in other steatohepatitis [36], wound healing [37], and hepatitis [38] studies, but has not been examined in anti-neuro-disorder research. In the current in vitro study, our results demonstrate that bee venom reversed ROS, MDA, SOD, and protein carbonyl production induced by Aβ_1–42_. To further explain these phenomena, we examined the effects of bee venom on the activation of the Nrf2/HO-1 signaling pathway. HO-1 is an intrinsic antioxidant enzyme that is considered the first line of cellular defense against oxidative damage [60]. Nrf2 is a transcription factor for HO-1. In the primitive stage, Nrf2 is locked within the Nrf2-Keap1 complex, and appropriate cellular protection stimulation can lead to the liberation of Nrf2 from the complex, thus promoting this transcription factor to migrate into the nucleus. Bee venom markedly upregulated the expression of Nrf2 in the nucleus, thus enhancing the overall production of HO-1 antioxidant enzyme activity, suggesting that the positive effect of bee venom on Aβ_1–42_-induced HT22 cell injury was attributed to its antioxidant properties. 

The TrkB/CREB/BDNF pathway is commonly studied due to its antioxidant properties, owing to its capacity to significantly reinforce Nrf2/HO-1 upregulation [40,52,57,59,61,62]. TrkB activation can stimulate the simultaneous upregulation of intracellular antioxidant defenses and brain-derived neurotrophic factor neuro-proliferative mechanisms [6]. In the current study, the presence of Aβ_1–42_ significantly depleted the TrkB/CREB/BDNF pathway. Remarkably, bee venom pre-treatment stimulated the production of p-TrkB and p-CREB, and in turn, increased the expression of BDNF [63]. Moreover, BDNF has been shown to bind to subsets of TrkB receptors, thus further upregulates neural proliferation and enhanced neurogenesis, increasing the neuronal cell’s ability to combat oxidative stress [64,65]. As mentioned, in previous studies of bee venom in cell models which seem to lack the significance of BDNF/TrkB/CREB, such as studies on steatohepatitis [36], wound healing [37], and hepatitis [38], bee venom also showed a remarkable ability to upregulate the Nrf2/HO-1 pathway. If this assumption is correct, it hints that bee venom could upregulate HO-1 production as the primary effect. However, it is unclear whether bee venom could directly enhance the TrkB/CREB/BDNF route itself, or if this effect is a result of cell redox stabilization thanks to maintained HO-1 levels. The recovery of this dominant neurotrophic not only ameliorates neuron cell metabolisms but also provides enhancement back to the Nrf2-ARE system through vibrant neurotrophic downstream signaling pathways that promote proliferation, such as the PI3K/Akt, MAPK (Ras/ref/Erk), and PLCγ (PKC or CaMK) [6]. Drug strategies targeting a single pathway usually do not achieve holistic results, and the evidence that bee venom enhances the cellular intrinsic antioxidative barrier as well as the neurotrophic system suggests that bee venom is an effective medicine for neurodegenerative disorders.

For in vivo research, the ICV injection of Aβ_1–42_ into mouse brains was established as a means of inducing oxidative stress and neuroinflammation, suppressing neurogenesis, and promoting cognitive impairments. This model depicts significant grades of Alzheimer’s progression symptoms and neurodegeneration results in general [13,14,15,16,17,18].

In this study, bee venom significantly restored the memory and learning capabilities of animals with cognitive impairment induced by Aβ. The neuroprocessing proficiency of mice is directly associated with hippocampal functioning, and Alzheimer’s disease patients also show signs of significant hippocampal dysfunction [66]. The hippocampal dentate gyrus is regarded as a key element in the development, recall, and prejudice of episodic memory and was, therefore, chosen for this study [53]. The significant increase in neurogenesis in this crucial hippocampal region proves the effect of bee venom at an anatomical scale.

The overexpression of oxidative stress level is closely related to pathological progression. To confirm the antioxidant effects of bee venom on the in vivo model, we measured ROS and MDA levels in the hippocampus and serum; these parameters were sharply increased due to Aβ_1–42_ ICV, and bee venom pre-treatment clearly decreased these oxidative stress markers. Bee venom also reduced the amount of NO buildup and iNOS protein expression in the hippocampus, which implies a reduction in neuronal-derived nitric oxide, a vital factor promoting neural diseases [67,68].

The balance of the cholinergic system is the foothold in cognitive function [69]. Previous studies have revealed that Alzheimer’s patients express significantly downregulated levels of mAChR 1; moreover, it is the main neuroreceptor in the cholinergic system [69]. Within the synaptic cleft, ACh attaches to postsynaptic mAChRs, and the synaptic apparatus is linked to the cyclic adenosine monophosphate/protein kinase A/CREB pathway. This crossing echoes the reality that the cholinergic system and neural neurotrophic factor signaling are reciprocal entities. The dysfunction of one can have a negative effect on the other, and they both synergistically facilitate the basis for brain cognitive tasks [70]. In our present study, bee venom significantly upregulated mAChR1 expression and regulated AChE and ACh levels, which were negatively disrupted by Aβ_1–42_. To confirm the effects of bee venom on neuron biochemistry, we assessed the intercellular neurotrophic factor pathway CREB/BDNF [71], which was upregulated by treatment with bee venom. This is in line with a previous study that showed that bee venom successfully recovered BDNF levels in a mouse model of vascular dementia [45]. Consistent with the in vitro experiments, our in vivo results show that bee venom-treated mice also expressed increased hippocampal p-CREB and BDNF levels, which suggests a significant recovery of brain neuron metabolism.

In this study, we exhibited bee venom to upregulate the Nrf2 into the nucleus and to upregulate HO-1 production, which, as mentioned, is a phenomenon observed by many researchers [36,37,38]. The explanation of the mechanisms by which bee venom upregulates Nrf2 is recommended for research in the future.

## 5. Conclusions

In neuro-aging, oxidative stress provoked by naturally deposited Aβ is thought to initiate neurodegenerative diseases and cognitive downfalls. The cellular antioxidant system is the first line of defense in many disorders, including neurodegeneration. Here, we revealed for the first time that the wide neuroprotective effects of bee venom are primarily possible due to its ability to promote Nrf2 nuclear translocation and increase HO-1 antioxidant enzyme production, a process that is also closely reinforced by the TrkB/CREB/BDNF pathway. This ability of bee venom to help regain cellular redox balance promotes its use as a holistic anti-neurodegeneration agent.

## Data Availability

The data presented in this study are available in the article.

## Abbreviations

Aβ, amyloid-beta; AIF, apoptosis-inducing factor; MDA, lipid peroxidation; iNOS, inducible nitric oxide synthase; MWM, Morris water maze; DCFDA, 2′,7′-dichlorodihydrofluorescein diacetate; Nrf2, nuclear factor erythroid 2-like 2; CytoC, cytochrome c; DAPI, 4′,6-diamidino-2-phenylindole; HO-1, heme oxygenase-1; M1, muscarinic acetylcholine receptors; SOD, superoxide dismutase; PI, propidium iodide; ROS: reactive oxygen species; TrkB, tropomyosin-related kinase receptor B; LDH, lactate dehydrogenase; AchE, acetylcholine esterase; Ach, acetylcholine; CleaCas3, cleaved caspase-3; NO, nitric oxide; CREB, cAMP response element-binding; mAchR 1, M1 muscarinic acetylcholine receptors; SC., subcutaneous injection; ICV, intracerebroventricular injection; RFP, red fluorescent protein; GFP, green fluorescent protein; p-, phosphorylated-; BDNF, brain-derived neurotrophic factor.
